# Pharmacokinetic Comparability of ABP 654, a Biosimilar to Ustekinumab, Administered Either via Prefilled Syringe or Autoinjector in Healthy Adults: Results from a Randomized, Open‐Label, Parallel‐Group Study

**DOI:** 10.1002/cpdd.70031

**Published:** 2026-02-05

**Authors:** Vincent Chow, Peter J. Winkle, Daniel T. Mytych, Jia Cao, Carolina Barragan, Alexander Colbert, Shalini Gupta, Waldemar Radziszewski, Janet Franklin

**Affiliations:** ^1^ Amgen Inc. Thousand Oaks CA USA; ^2^ Anaheim Clinical Trials Anaheim CA USA

**Keywords:** autoinjector, bioequivalence, biosimilar, pharmacokinetics, ustekinumab

## Abstract

ABP 654, a biosimilar to ustekinumab reference product, is available in a prefilled syringe (PFS) for subcutaneous (SC) use. The ABP 654 autoinjector pen (AIP) has recently been developed with an aim to improve the injection experience for patients and caregivers. This study was designed to assess the similarity of pharmacokinetics (PK), safety, and immunogenicity of a 90‐mg SC injection of ABP 654 administered via PFS or AIP in healthy volunteers. A total of 156 adults were randomized at a ratio of 1:1. PK bioequivalence was established between the PFS and AIP groups based on the 90% CIs of the geometric mean ratios for the primary PK endpoints of maximum observed serum concentration (C_max_) and area under the serum concentration–time curve from time 0 extrapolated to infinity (AUC_inf_) being contained within the prespecified margin of 0.8 to 1.25. The frequency, type, and severity of adverse events as well as the incidence of antidrug antibodies were similar between the PFS and AIP groups. Overall, the results support a conclusion of PK bioequivalency as well as comparable safety and immunogenicity of ABP 654 administered via PFS and AIP.

WEZENLA/WEZLANA (ustekinumab‐auub, ABP 654; Amgen Inc., Thousand Oaks, CA) is a biosimilar to STELARA (ustekinumab; Janssen Biotech, Inc. Horsham, PA), referred to hereafter as ABP 654, used in the management of various inflammatory conditions, including moderate‐to‐severe plaque psoriasis.^1^, ^2^, ^3^, ^4^, ^5^, ^6^, ^7^ A biosimilar is highly similar to and has no clinically meaningful differences from a licensed biologic drug, often called the reference product (RP).^8^, ^9^ Development of ABP 654 began with an analytical characterization comparing its structural and functional characteristics to ustekinumab RP approved in the United States (ustekinumab US) and European Union (ustekinumab EU). An extensive array of sophisticated analytical tests demonstrated that ABP 654, ustekinumab US, and ustekinumab EU have the same amino acid sequence, and that they have comparable structures, biologic activity, and purity.^10^ Moreover, results from a randomized, double‐blind study in healthy volunteers exhibited similar pharmacokinetic (PK), safety, and immunogenicity profiles following a single dose of ABP 654, ustekinumab US, or ustekinumab EU.^11^ In addition, ABP 654 and ustekinumab RP were found to be comparable in efficacy, safety, and immunogenicity in a randomized, double‐blinded comparative clinical trial over the course of 52 weeks in patients with moderate‐to‐severe plaque psoriasis.^12^

ABP 654 first became available in a prefilled syringe (PFS) for subcutaneous (SC) injection. To expand treatment options and improve the injection experience for patients and caregivers, an ABP 654 autoinjector pen (AIP; ConfiPen), which is preassembled with a PFS, has now been approved as a single‐use, handheld, latex‐free, mechanical syringe. Here, we report the results of a randomized, open‐label, parallel‐group clinical trial in healthy adults designed to assess the similarity in PK, safety, and immunogenicity of a single ABP 654 SC injection administered via PFS or AIP.

## Methods

### Study Design and Participant Population

This study complied with International Council for Harmonization Good Clinical Practice guidelines and was conducted in accordance with the principles expressed in the Declaration of Helsinki. The protocol, informed consent form, and materials provided to study participants were approved by a central Institutional Review Board (Advarra IRB; Columbia, MD). Investigators were responsible for obtaining written informed consent from participants after explanation of the aims, methods, anticipated benefits, and potential hazards of the study.

The study, conducted at two centers in the United States, enrolled healthy male and female adults 18 to 45 years old, with a body weight between 50 and 90 kg and a body mass index between 18 and 30 kg/m^2^ at the time of screening. Study participants were to have a normal or clinically acceptable physical examination, laboratory test results, vital signs, and 12‐lead electrocardiogram (ECG). In addition, the use of concomitant medications that could affect study assessments was restricted per protocol during screening and throughout the study, with routine, non‐interfering medications allowed at the investigator's discretion. Study participants were not to receive or have previously received other investigational drugs (or be currently using an investigational device) within 30 days or 5 half‐lives of the investigational drugs (whichever was longer) prior to randomization and no prior exposure to ABP 654 (or any other biosimilar to ustekinumab), ustekinumab, IL‐23 antagonists, IL‐12 antagonists, or related compounds. Participants were excluded from the study if they were pregnant, nursing, or planning to conceive.

Approximately 154 (n = 77 per treatment group) study participants were planned to provide >90% power to establish equivalence of the primary PK endpoints based on the assumptions of between‐subject variability (as measured by coefficient of variation [CV]) of 40% for ABP 654 PFS and AIP, a true geometric mean ratio (GMR) of 1 between ABP 654 PFS and AIP, a bioequivalence margin of 0.8 to 1.25, a 5% dropout rate, and two 1‐sided tests at α = 0.05.

ABP 654 was supplied in a PFS and in an AIP containing 90 mg/mL. The PFS consisted of a 1 mL Type 1 glass syringe barrel with a staked 27‐gauge fixed 1/2 inch needle, an elastomeric plunger, and a non‐rigid needle shield. Each 1 mL PFS delivered 90 mg ABP 654, L‐histidine and L‐histidine hydrochloride monohydrate (1 mg), polysorbate 80 (0.04 mg), and sucrose (76 mg), pH of 6.0. The ABP 654 AIP was a single‐use, handheld, preservative‐ and latex‐free, mechanical (spring‐driven) injection device that was provided ready‐to‐use and was preassembled with a PFS. The syringe consisted of a 1 mL Type 1 glass syringe barrel with a staked 27‐gauge fixed 1/2 inch needle, an elastomeric plunger, and a rigid needle shield. Identical to the ABP 654 PFS presentation, each 1 mL ABP 654 AIP delivered 90 mg ABP 654, L‐histidine and L‐histidine hydrochloride monohydrate (1 mg), polysorbate 80 (0.04 mg), and sucrose (76 mg), pH of 6.0.

Prior to dosing, study participants were randomized 1:1 through an interactive voice/web response system to receive a 90‐mg dose of ABP 654 administered either via PFS or AIP. Randomization was stratified by gender. Screening occurred within day −28 to day −2 before dosing. Following screening, study participants were admitted to the Clinical Pharmacology Unit (CPU) on day −1. A 90‐mg SC injection was selected as it is the highest approved dose based on the product labeling for ustekinumab RP^1^, ^2^ and in consideration of the fact that it is widely used in clinical practice. In addition, the 90‐mg dose falls within the linear dose–exposure range of 24 to 240 mg.^2^ The 112‐day PK sampling duration of the study represents more than 4.5 times the terminal elimination half‐life (t_1/2_; i.e., 22.1 days for healthy volunteers^13^) of ustekinumab RP and is expected to provide more than 80% of the area under the serum concentration–time curve (AUC) from time 0 extrapolated to infinity (AUC_inf_) in over 90% of study participants.

Dosing occurred on day 1 after baseline procedures were completed. Study participants remained in the CPU until day 3 for PK and safety evaluations. Participants visited the CPU on days 7, 9, 11, 13, 21, 28, 35, 49, 56, 70, 98, and 112 (end‐of‐study [EOS] visit) for PK, safety, and immunogenicity evaluations. Study personnel surveyed study participants for potential adverse events each day after their discharge until day 15.

### Pharmacokinetic Analyses

The primary objective of the study was to investigate the equivalence, based on exposure (maximum observed serum concentration [C_max_] and AUC_inf_) of ABP 654 after a single SC injection delivered by PFS or AIP in healthy volunteers. Secondary PK endpoints included AUC from time 0 to the last quantifiable concentration (AUC_last_), time at which C_max_ is observed (T_max_), and t_1/2_.

Concentrations of ABP 654 were measured using a sensitive and validated electrochemiluminescent assay (Meso Scale Diagnostics LLC, Rockville, MD) that uses a pair of anti‐idiotype monoclonal antibodies to reliably capture and detect ABP 654 at a range of 20–32,000 ng/mL.^14^, ^15^, ^16^

Serum concentrations of ABP 654 were summarized descriptively by treatment and timepoint using the PK concentration analysis set (study participants who received any amount of ABP 654 and reported at least one serum concentration).

Early termination measurements were assigned to the closest scheduled time for purposes of grouping and summaries of PK concentrations. Serum PK parameters for ABP 654 were estimated using noncompartmental methods with WinNonlin using best‐fit regression. PK parameters were determined from the concentration–time profiles, and AUCs were calculated using the linear trapezoidal method. Actual sampling times were used in all calculations. If the actual time was missing, the scheduled time was to be substituted and flagged.

PK endpoints were summarized descriptively using the PK parameter analysis set (study participants from the PK concentration analysis set with an evaluable ABP 654 serum concentration–time profile). The point estimates and 90% CIs for GMR of PK endpoints (AUC_inf_, C_max_, and AUC_last_) were calculated from an analysis of covariance model adjusted for baseline weight using the PK parameter analysis set. Prior to statistical modeling, PK parameters were log_e_ transformed. Point estimates and 90% CIs for the mean difference in logarithmic PK parameters were estimated for comparison of ABP 654 AIP versus ABP 654 PFS. The point estimates and 90% CIs for GMR were then calculated by transforming back to the original scale.

PK equivalence was to be concluded if the 90% CIs for the GMR between ABP 654 AIP and ABP 654 PFS for AUC_inf_ and C_max_ fell entirely within the prespecified bioequivalence margin of 0.8 to 1.25. A similar analysis was performed for AUC_last_.

### Safety Analyses

Safety endpoints were summarized descriptively using the safety analysis set (study participants who received a dose of ABP 654). Adverse events were graded according to Common Terminology Criteria for Adverse Events (CTCAE) version 4.03. Unless otherwise noted, the term “adverse event” refers to a treatment‐emergent adverse event.

All reported adverse events, including Grade ≥3 adverse events, any fatal adverse events, any serious adverse events, any adverse events leading to discontinuation from study, and any events of interest, were coded to the appropriate system organ class and preferred term according to MedDRA version 24.1 and summarized.

### Immunogenicity Analyses

Binding and neutralizing antidrug antibodies (ADAs) were tabulated by nominal visit and treatment using the safety analysis set. Total ADA incidence was defined as the number of participants with an on‐study result and a positive ADA result at any time divided by the number of participants with an on‐study result. Pre‐existing ADA incidence was defined as the number of participants with a positive ADA result at or before baseline divided by the number of participants with a result at baseline. Developing ADA incidence through day 11, through day 35, and through EOS was defined as the number of participants with a negative or absent ADA result at baseline and a positive antibody result at a postbaseline visit through the respective period and was divided by the number of participants with at least one postbaseline result within the respective period. Treatment‐boosted ADA incidence was defined as the number of participants who were binding ADA positive at baseline with a ≥4× increase postbaseline within the respective period divided by the number of participants with at least one postbaseline result within the respective period. Transient ADA incidence was summarized through EOS and was defined as the number of participants who had a negative or absent ADA result at baseline and a positive ADA result at a postbaseline visit (including unscheduled visit) with a negative result at the participant's last timepoint tested through EOS divided by the number of participants with at least one postbaseline result through the EOS.

## Results

### Demographic and Other Baseline Characteristics

Demographics and baseline characteristics were similar between the ABP 654 PFS and ABP 654 AIP groups (Table 1). Overall, 51.9% were female at birth, 59.6% were white, and 57.1% were not Hispanic or Latino. The mean (SD) age was 32.1 (7.1) years with a range of 18 to 45 years.

**Table 1:** Demographic and Baseline Characteristics

	ABP 654 PFS (N = 78)	ABP 654 AIP (N = 78)	Total (N = 156)
Sex at birth, n (%)			
Female	40 (51.3)	41 (52.6)	81 (51.9)
Male	38 (48.7)	37 (47.4)	75 (48.1)
Race, n (%)			
White	45 (57.7)	48 (61.5)	93 (59.6)
Black or African American	25 (32.1)	24 (30.8)	49 (31.4)
Asian	4 (5.1)	5 (6.4)	9 (5.8)
Multiple‐White, Black or African American, Asian	3 (3.8)	1 (1.3)	4 (2.6)
American Indian or Alaska Native	1 (1.3)	0 (0.0)	1 (0.6)
Ethnicity, n (%)			
Hispanic or Latino	30 (38.5)	36 (46.2)	66 (42.3)
Not Hispanic or Latino	47 (60.3)	42 (53.8)	89 (57.1)
Not reported	1 (1.3)	0 (0.0)	1 (0.6)
Mean age (years, SD)	32.3 (7.4)	31.8 (6.9)	32.1 (7.1)
Mean weight (kg, SD)	72.5 (8. 9)	72.2 (9.3)	72.3 (9.1)
Mean BMI (kg/m^2^, SD)	25.4 (2.6)	25.7 (2.5)	25.6 (2.6)

AIP, autoinjector pen; BMI, body mass index; PFS, prefilled syringe.

### Pharmacokinetics

**Figure 1:**
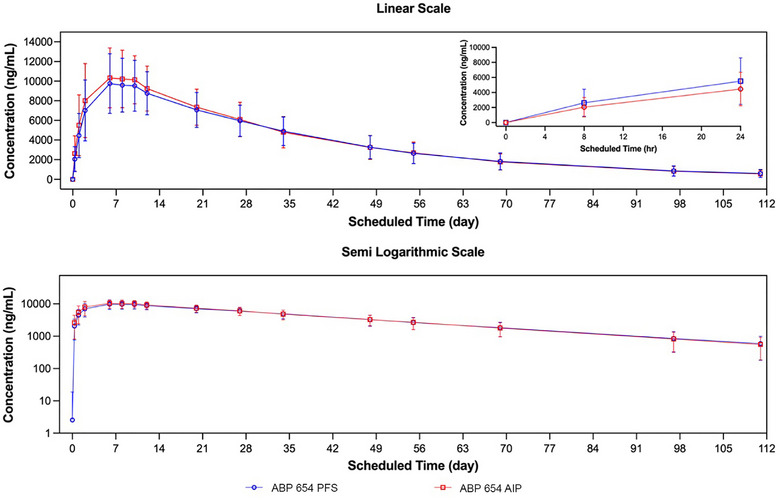
Mean (± SD) PK concentrations over time by treatment. AIP, autoinjector pen; PFS, prefilled syringe; PK, pharmacokinetic. n = 78 for the ABP 654 PFS treatment group; n = 78 for the ABP 654 AIP treatment group.

Mean (±SD) serum ABP 654 PFS and ABP 654 AIP concentration–time profiles were similar over the entire course of sampling (Figure 1). For the comparisons of ABP 654 administered via PFS to ABP 654 administered via AIP, the point estimates and 90% CIs of the GMR fell within the prespecified margin of 0.8 to 1.25 for both the primary PK endpoints (AUC_inf_ and C_max_) and the secondary PK endpoint of AUC_last_ (Table 2). Therefore, PK equivalence was demonstrated between ABP 654 administered via PFS and AIP.

**Table 2:** Primary Analysis of PK Parameters

Treatment and Comparison	AUC_inf_ (h·µg/mL) LS Geometric Mean [n]	C_max_ (µg/mL) LS Geometric Mean [n]	AUC_last_ (h·µg/mL) LS Geometric Mean [n]
ABP 654 PFS	9654 [74]	9.8 [76]	9049 [76]
ABP 654 AIP	9720 [77]	10.3 [78]	9285 [78]
**Ratio of LS Geometric Means (90% CI)**			
ABP 654 AIP/ABP 654 PFS	1.01 (0.92, 1.10)	1.05 (0.97, 1.14)	1.03 (0.94, 1.12)

AIP, autoinjector pen; AUC, area under the curve; AUC_inf_, AUC from time 0 extrapolated to infinity; AUC_last_, AUC from time 0 to the last quantifiable concentration; CI, confidence interval; C_max_, maximum observed serum concentration; h, hour; LS, least squares; PFS, prefilled syringe; PK, pharmacokinetic.

Additional PK parameters and descriptive statistics are provided in Table 3. In general, the geometric means of individual PK parameters were comparable following a 90‐mg dose of ABP 654 via PFS and AIP. Exposure was similar between the two groups. Peak ABP 654 concentrations were recorded on average about 8 days after injection. After achieving peak ABP 654 concentrations, levels fell in a monophasic manner. The estimated t_1/2_ was about 23 days for both groups. The mean percentage of AUC_inf_ due to extrapolation from the last quantifiable concentration observed to infinity (AUC_%Extrap_) was 5.1% and 4.5% for the PFS and AIP groups, respectively, indicating that the duration of PK sampling was adequate for AUC_inf_ assessment.

**Table 3:** Summary of Key ABP 654 PFS and ABP 654 AIP PK Parameters

Treatment	C_max_ (µg/mL) GM (Geo CV [%])	T_max_ (h) Median (Range)	AUC_last_ (h·µg/mL) GM (Geo CV [%])	AUC_inf_ (h·µg/mL) GM (Geo CV [%])	t_1/2_ (h) Mean (SD)	AUC_%Extrap_ (%) Mean (SD)	AUC_%Extrap_ (%) Range
ABP 654 PFS	9.8 (31.7)	191.9 (48.0, 599.6)	9040 (35.1)	9660 (36.6)	575.8 (155.4)	5.1 (3.5)	0.1, 14.4
ABP 654 AIP	10.3 (32.8)	191.1 (48.0, 291.9)	9300 (34.8)	9720 (36.8)	556.1 (143.3)	4.5 (2.9)	0.2, 11.9

Geometric mean and Geo CV were only calculated for values >0.

AIP, autoinjector pen; AUC, area under the curve; AUC_%Extrap_, percentage of AUC_inf_ due to extrapolation from the last quantifiable concentration observed to infinity; AUC_inf_, AUC from time 0 extrapolated to infinity; AUC_last_, AUC from time 0 to the last quantifiable concentration; C_max_, maximum observed serum concentration; Geo CV, geometric coefficient of variation; GM, geometric mean; h, hour; PFS, prefilled syringe; PK, pharmacokinetic; t_1/2_, terminal elimination half‐life; T_max_, time at which C_max_ is observed.

### Safety

A summary of adverse events is shown in Table 4. Most adverse events were CTCAE Grade 1 or 2 in severity. Adverse events were reported by 24 (30.8%) study participants in the ABP 654 PFS treatment group and 24 (30.8%) in the ABP 654 AIP treatment group. Across treatment groups, the most commonly reported adverse event was headache (11 [14.1%] study participants in the ABP 654 PFS treatment group and 3 [3.8%] in the ABP 654 AIP treatment group).

**Table 4:** Summary of Adverse Events

	ABP 654 PFS (N = 78) n (%)	ABP 654 AIP (N = 78) n (%)
Any adverse event	24 (30.8)	24 (30.8)
Any Grade ≥3 adverse event	0 (0.0)	2 (2.6)
Any fatal adverse event	0 (0.0)	0 (0.0)
Any serious adverse event	0 (0.0)	2 (2.6)
Any adverse event leading to discontinuation of study	0 (0.0)	0 (0.0)
Any adverse event of interest	1 (1.3)	1 (1.3)
Any COVID‐19 adverse event^a^	3 (3.8)	1 (1.3)
Adverse events experienced by ≥2 study participants by preferred term		
Headache	11 (14.1)	3 (3.8)
Diarrhea	5 (6.4)	3 (3.8)
COVID‐19	3 (3.8)	1 (1.3)
Dizziness	3 (3.8)	0 (0.0)
Nausea	3 (3.8)	1 (1.3)
Abdominal discomfort	2 (2.6)	0 (0.0)
Constipation	2 (2.6)	1 (1.3)
Nasopharyngitis	2 (2.6)	1 (1.3)
Aspartate aminotransferase increased	1 (1.3)	2 (2.6)
Alanine aminotransferase increased	0 (0.0)	2 (2.6)
Erythema	0 (0.0)	2 (2.6)

Only treatment‐emergent adverse events were summarized. For each category and preferred term, study participants were included only once, even if they experienced multiple events in that preferred term.

AIP, autoinjector pen; COVID‐19, coronavirus disease 2019; eCRF, electronic case report form; PFS, prefilled syringe.

^a^ Adverse event data collected on the adverse events eCRF was used to identify COVID‐19 adverse events using the COVID‐19 Standardized MedDRA Query narrow search strategy.

Serious (Grade ≥3) adverse events were not reported in any study participant in the ABP 654 PFS treatment group and were reported in 2 (2.6%) in the ABP 654 AIP treatment group (one study participant experienced appendicitis and sepsis, and one experienced a combined tibia–fibula fracture; these events were unrelated to ABP 654 according to the investigator). No study participant experienced a fatal (Grade 5) adverse event.

### Immunogenicity

ADA results are shown in Table 5. All study participants had at least one ADA result. Two study participants (2.6%) in the ABP 654 PFS treatment group and none in the ABP 654 AIP treatment group tested positive for pre‐existing binding ADAs at or before baseline; one study participant (1.3%) in the ABP 654 PFS treatment group tested positive for pre‐existing neutralizing ADAs at or before baseline.

**Table 5:** Antidrug Antibody Results

	ABP 654 PFS (N = 78)	ABP 654 AIP (N = 78)
Participants with an on‐study result^a^	78	78
Total antibody incidence, n (%)		
Binding antibody positive anytime	12 (15.4)	16 (20.5)
Neutralizing antibody positive anytime	7 (9.0)	6 (7.7)
Participants with a result at baseline	78	78
Pre‐existing antibody incidence, n (%)		
Binding antibody positive at or before baseline	2 (2.6)	0 (0.0)
Neutralizing antibody positive at or before baseline	1 (1.3)	0 (0.0)
Participants with a postbaseline result through day 11	73	76
Treatment‐boosted antibody incidence, n (%)		
Binding antibody positive at baseline with a ≥4× increase in magnitude postbaseline	1 (1.4)	0 (0.0)
Developing antibody incidence, n (%)		
Binding antibody positive postbaseline with a negative or no result at baseline	5 (6.8)	4 (5.3)
Neutralizing antibody positive postbaseline with a negative or no result at baseline	4 (5.5)	3 (3.9)
Participants with a postbaseline result through day 35	78	78
Treatment‐boosted antibody incidence, n (%)		
Binding antibody positive at baseline with a ≥4× increase in magnitude postbaseline	1 (1.3)	0 (0.0)
Developing antibody incidence, n (%)		
Binding antibody positive postbaseline with a negative or no result at baseline	5 (6.4)	10 (12.8)
Neutralizing antibody positive postbaseline with a negative or no result at baseline	4 (5.1)	4 (5.1)
Participants with a postbaseline result through EOS	78	78
Treatment boosted antibody incidence, n (%)		
Binding antibody positive at baseline with a ≥4× increase in magnitude postbaseline	1 (1.3)	0 (0.0)
Developing antibody incidence, n (%)		
Binding antibody positive postbaseline with a negative or no result at baseline	10 (12.8)	16 (20.5)
Transient^b^	2 (2.6)	5 (6.4)
Neutralizing antibody positive postbaseline with a negative or no result at baseline	6 (7.7)	6 (7.7)
Transient^b^	3 (3.8)	3 (3.8)

Baseline was defined as the last non‐missing assessment taken prior to the first dose of study investigational product. Percentages were calculated using the corresponding category count as the denominator.

AIP, autoinjector pen; EOS, end‐of‐study; PFS, prefilled syringe.

^a^ Participants considered on‐study after signing informed consent.

^b^ Negative result at the participant's last time point tested within the study period.

All study participants had at least one postbaseline result through the EOS. Of the study participants with a postbaseline result through EOS, 10 (12.8%) in the ABP 654 PFS group and 16 (20.5%) in the ABP 654 AIP group were positive for binding ADAs (Table 5). Of these, the results were transient (i.e., negative results at the participant's last time point tested within the study period) for 2 (2.6%) and 5 (6.4%) study participants, respectively. Of the study participants with a postbaseline result through EOS, 6 (7.7%) in the ABP 654 PFS group and 6 (7.7%) in the ABP 654 AIP group were positive for neutralizing ADAs. Within these groups, the results were transient for 3 (3.8%) and 3 (3.8%) study participants, respectively. Of study participants with a postbaseline result through the EOS, 1 (1.3%) in the ABP 654 PFS group and none in the ABP 654 AIP group were positive for treatment‐boosted ADAs (i.e., binding ADA positive at baseline with a ≥4× increase postbaseline).

## Discussion

PK equivalence was established between the ABP 654 PFS and ABP 654 AIP groups based on the 90% CIs of the GMR for the primary PK endpoints of AUC_inf_ and C_max_ being contained within the prespecified margin of 0.8 to 1.25. Results from analysis of the key secondary PK endpoints (AUC_last_, T_max_, and t_1/2_) also demonstrated PK similarity between the ABP 654 PFS and ABP 654 AIP groups. In addition, adverse events were comparable between treatment groups and were consistent with the safety profile of the RP. Headache (11 [14.1%] and 3 [3.8%] study participants, respectively) was the only adverse event with at least a 5% difference between the treatment groups by preferred term. Despite a slight numerical difference in binding ADAs of ABP 654 administered via PFS versus ABP 654 administered via AIP, a lack of clinical impact relating to this difference is supported by the demonstration of PK equivalence and comparable safety profiles between PFS and AIP treatment groups.

The PK equivalence of ABP 654 and ustekinumab RP was previously demonstrated in healthy volunteers.^11^ Because different administration devices may impact the bioavailability of a drug, the current study aimed to determine if the AIP delivers a dose of ABP 654 that results in similar PK, safety, and immunogenicity as would be obtained when using the PFS. Healthy volunteers were chosen for this study because they provide a homogenous population in which to compare the PK of ABP 654 administered by the two devices. In contrast, a study in patients would be associated with potential confounding variables, including underlying disease and concomitant medications.

An autoinjector may increase patient adherence to treatment, quality of life, and satisfaction.^17^, ^18^, ^19^, ^20^, ^21^, ^22^ It may also be easier to use in comparison to a PFS and lessen the burden of clinic visits. The syringe in the ABP 654 AIP was the same as the ABP 654 PFS with the following two exceptions: (1) the composition of the syringe needle shield in the AIP was changed to enable elimination of latex and features a rigid outer cover that helps lessen potential needle phobia,^23^, ^24^ and (2) the syringe in the AIP did not include the plunger rod. Both the PFS and AIP have the same needle length and diameter and are designed to deliver an SC injection with the same volume and needle depth.

## Conclusions

An AIP has been developed to improve the self‐injection experience for patients taking ABP 654. Overall, the results support conclusions of PK bioequivalency as well as comparable safety and immunogenicity profiles of ABP 654 administered via PFS and AIP. Based on these results and previous studies demonstrating the structural, functional, PK, and clinical similarity of ABP 654 and ustekinumab RP, it is anticipated that ABP 654 delivered via AIP will perform comparably to ABP 654 or ustekinumab RP administered by PFS.

## Conflicts of Interest

Vincent Chow, Daniel T. Mytych, Jia Cao, Carolina Barragan, Alexander Colbert, Shalini Gupta, Waldemar Radziszewski, and Janet Franklin are/were employees of Amgen and hold Amgen stock at the time of the study. Peter J. Winkle declares no competing interests.

## Funding

This study and article processing charges were funded by Amgen Inc., Thousand Oaks, CA.

In general, Amgen does not grant external requests for individual patient data for the purpose of reevaluating safety and efficacy issues already addressed in the product labeling. A committee of internal advisors review requests. If not approved, a Data Sharing Independent Review Panel may arbitrate and make the final decision. Requests that pose a potential conflict of interest or an actual or potential competitive risk may be declined at Amgen's sole discretion and without further arbitration. Upon approval, information necessary to address the research question will be provided under the terms of a data sharing agreement. This may include anonymized individual patient data and/or available supporting documents, containing fragments of analysis code were provided in analysis specifications. Further details are available at the following: http://www.amgen.com/datasharing

## Data Availability

There is a plan to share data. This may include de‐identified individual patient data for variables necessary to address the specific research question in an approved data sharing request; also related data dictionaries, study protocol, statistical analysis plan, informed consent form, and/or clinical study report. Data sharing requests relating to data in this manuscript will be considered after the publication date and (1) this product and indication (or other new use) have been granted marketing authorization in both the United States and Europe, or (2) clinical development discontinues and the data will not be submitted to regulatory authorities. There is no end date for eligibility to submit a data sharing request for these data. Qualified researchers may submit a request containing the research objectives, the Amgen product(s) and Amgen study/studies in scope, endpoints/outcomes of interest, statistical analysis plan, data requirements, publication plan, and qualifications of the researcher(s).
